# The Central Illinois Medical Society

**Published:** 1873-08-01

**Authors:** W. G. Cochran

**Affiliations:** Sec’y.; Champaign, Ill.


### Society Reports.

### THE CENTRAL ILLINOIS MEDICAL SOCIETY.

CHAMPAIGN, ILL., June 10, 1873.

The Central Illinois Medical Society met in regular session at Champaign, June 10th, 1873.

Society was called to order by Dr. J. Wright, President.

Minutes of preceding meeting read and approved.

Dr. S. H. Birney, Chairman of Committee of Arrangements, extended to the Society a thrice hearty welcome, to which Dr. Laughlin, on the part of the Society, responded, feeling that, under such favorable circumstances, the Society would enjoy a pleasant session.

A Nominating Committee, consisting of one member from each county, was appointed.

Dr. J. Denning, of St. Joseph, Dr. E. C. Bartholon, of Mahomet, and Drs. Jas. Bartholon and J. D. Mandeville, of Philo, were recommended for membership. Censors reporting favorably, they were duly elected.

Nominating Committee reported, for President, Dr. Wm. Hill, Bloomington; 1st Vice-President, Dr. J. D. Gardner, Mahomet; 2d Vice-President, Dr. Z. H. Madden, Clinton; Secretary, Dr. W. G. Cochrane, Farmer City; Corresponding Secretary, Dr. R. G. Laughlin, Bloomington; Treasurer, Dr. H. C. Howard, Champaign; Censors, Dr. S. H. Birney, Urbana; Dr. C. T. Orner, Saybrook; Dr. J. S. Miller, Farmer City; Dr. C. Goodbrake, Clinton; Dr. J. Little, Le Roy.

The report was accepted, and officers duly elected, as recommended by Nominating Committee.

Dr. W. Hill, on taking the chair, thanked the Society for the high honor conferred on him.

Dr. Wright, the retiring President, delivered his valedictory address, which was received with much applause; and, on motion, the Doctor was requested to furnish copies for publication in Chicago and Cincinnati medical journals; also in the different county papers throughout the district.

Dr. Birney offered the following resolution, which was adopted by sections:

*Resolved, first*, That Woodford, Livingstone, Tazewell, Iroquois, McLean, Ford, Vermilion, De Witt, Champaign, Piatt, Macon, Mason, and Logan counties, for the present, constitute the boundaries of the Society.

*Resolved second*, That each county be earnestly requested — where they do not already exist — to at once organize County Medical Societies.

*Resolved, third*, That each county be requested to send delegates to the meetings of this Society.

On motion, adjourned until half-past seven, P. M.

Evening session called to order by the President, Dr. Hill.

Reports of Special Committes being in order, Dr. S. H. Birney read a very interesting report, prepared by Dr. W. Hill, of Bloomington, on Inguinal Hernia, and its Reduction by Dilitation of the Ring with the Finger, and Report of Cases. This being a new mode of reducing this hernia, quite a spirited discussion ensued. The Doctor was well-prepared to defend his new theory; and the Society has a reason to believe it will be favorably received by the medical profession.

Dr. J. T. Pearman, Chairman of Committee on Practical Medicine, made a verbal report of a very interesting case.

Dr. M. S. Brown, Chairman of Committee on Obstetrics and Diseases of Women, made a very interesting report, his subject being a very interesting one. It elicited a lively discussion.

The President announced the following special committees:

*Surgery.*—Dr. S. H. Birney, Urbana, Chairman; Dr. H. C. Luce, Bloomington, and Dr. J. Little, Le Roy.

*Practical Medicine.*—Dr. R. G. Laughlin, Bloomington, Chairman; Dr. J. T. Pearman, Champaign; Dr. J. K. Edmiston, Clinton.

*Obstetrics and Diseases of Women.*—Dr. W. G. Cochran, Farmer City, Chairman; Dr. J. Wright, Clinton, and Dr. C. F. Orner, Saybrook.

*Pathology.*—Dr. E. A. Kratz, Champaign.

*Microscopy.*—Dr. R. Huddleston, Champaign.

*Eye and Ear.*—Drs. Jno. Clouser, Farmer City, and N. B. Cole, Bloomington.

Dr. R. G. Laughlin offered the following resolution, which was adopted:

*Resolved*, That competitive bidding for the practice of hospitals, county poor-houses, and other public institutions, is wrong in principle, contrary to the ethics governing the legitimate practice of medicine, and degrading to the dignity of a regular member of our honorable profession.

On motion, adjourned until half past seven A. M. June 11th.

SECOND DAY.

Second day's session called to order by the President, Dr. Wm. Hill.

Dr. Howard was appointed to report on Hypodermic Injections.

Dr. Pearman was appointed to report on Skin Grafting.

Dr. J. Wright was appointed to report on Puerperal Convulsions.

Dr. Cochrane moved, that when the Society adjourned, it adjourn to meet in Clinton, the second Tuesday in December next. Carried.

The Chair appointed as a Committee of Arrangement, Drs. J. Wright, of Clinton; C. Goodbrake, Clinton, and T. K. Edmiston.

On motion of Dr. Wright, the Secretary was directed to furnish copies of Proceedings for publication in Chicago and Cincinnati medical journals.

On motion of Dr. Orner, the Society adjourned.

W. G. COCHRAN, M.D., *Sec'y.*

